# Development of Bipolar All-solid-state Lithium Battery Based on Quasi-solid-state Electrolyte Containing Tetraglyme-LiTFSA Equimolar Complex

**DOI:** 10.1038/srep08869

**Published:** 2015-03-09

**Authors:** Yoshiyuki Gambe, Yan Sun, Itaru Honma

**Affiliations:** 1 Institute of Multidisciplinary Research for Advanced Materials, Tohoku University, 2-1-1 KatahiraAoba-ku, Sendai, Miyagi 980-8577, Japan

## Abstract

The development of high energy–density lithium-ion secondary batteries as storage batteries in vehicles is attracting increasing attention. In this study, high-voltage bipolar stacked batteries with a quasi-solid-state electrolyte containing a Li-Glyme complex were prepared, and the performance of the device was evaluated. Via the successful production of double-layered and triple-layered high-voltage devices, it was confirmed that these stacked batteries operated properly without any internal short-circuits of a single cell within the package: Their plateau potentials (6.7 and 10.0 V, respectively) were two and three times that (3.4 V) of the single-layered device, respectively. Further, the double-layered device showed a capacity retention of 99% on the 200th cycle at 0.5 C, which is an indication of good cycling properties. These results suggest that bipolar stacked batteries with a quasi-solid-state electrolyte containing a Li-Glyme complex could readily produce a high voltage of 10 V.

Lithium-ion secondary batteries are expected to be applied as high energy–density devices for large-scale uses such as electric vehicles^1^,^2^. However, commercially available lithium-ion secondary batteries are at risk for liquid leakage and ignition because of the use of organic liquid electrolytes. Therefore, it is necessary to improve their safety. To fulfill this requirement, all-solid-state lithium-ion secondary batteries with non-flammable solid electrolytes are attracting attention. As shown in Figure 1, the use of solid electrolytes enables the stacking of multiple electric cells in series within a single package^3^,^4^,^5^; this allows us to reduce the weight of the package and attain a higher energy density as compared to a single-layered cell. However, the availability of materials is limited to solid electrolytes that have high lithium-ion conductivity and are stable towards lithium metals^6^,^7^,^8^. Further, there have been reports that suggested that it is difficult to form a good interface between cathode active materials and electrolytes when using solid electrolytes^9^. Therefore, the development of novel solid electrolyte materials that can resolve these problems is required.

Ionic liquids are expected to be high-performance electrolyte materials because of their high conductivity, wide potential window, low vapor pressure and flame resistance, etc^10^,^11^,^12^. In recent years, Watanabe's research group has proposed novel complex-type ionic liquids and reported the proper operation of devices containing tetraethylene glycol dimethyl ether (Tetraglyme, G4) and Li-TFSA, i.e., ether-type solvents with an equimolar complex^13^,^14^,^15^,^16^,^17^,^18^. Ionic liquids can be handled as quasi-solid-states or gels because of their interactions with oxides^19^,^20^,^21^,^22^,^23^,^24^,^25^,^26^ and are being studied for energy-device applications such as dye-sensitized solar cells^20^ and lithium-ion secondary batteries^22^,^23^,^24^,^25^,^26^. Our research group has developed solid electrolytes that could be handled as quasi-solid-states because of the strong interactions between the ionic liquids and oxide nanoparticles; these materials showed similar transport properties as bulk ionic liquids. We also investigated the quasi-solidification of the Li-Glyme complex as an ionic liquid complex; an all-solid-state lithium battery with this electrolyte was proven to operate properly^25^,^26^ and are for the preliminarily tested bipolar multilayered device in our previous reports^27^. Therefore, in this study, we prepared high-voltage bipolar stacked batteries using a quasi-solid-state electrolyte containing a Li-Glyme complex, and the performance of this device was evaluated.

## Results

### Evaluation of bipolar stacked all-solid-state Li battery

**Figure 2:**
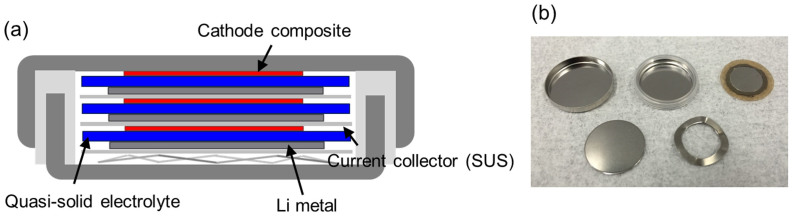
(a) Structure of the device of a triple-layered bipolar stacked all-solid-state Li battery and (b) photograph of the components of a bipolar stacked cell.

**Figure 3:**
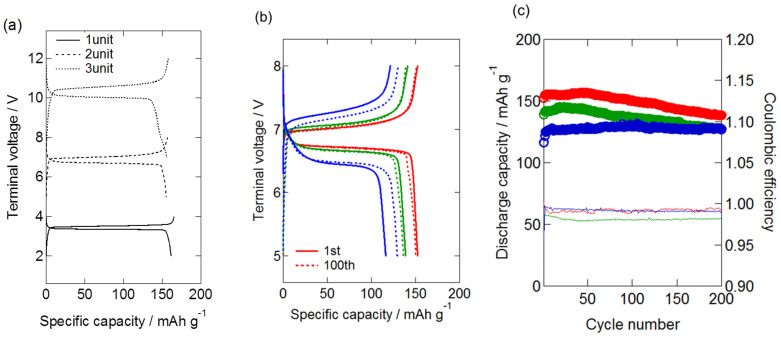
(a) 10^th^ charge–discharge profiles of bipolar stacked all-solid-state Li batteries containing one, two and three cell units at the C-rate of 0.1 C. (b) 1^st^ and 100^th^ charge-discharge profiles and (c) cycling properties of double-layered all-solid-state Li batteries at 35°C at 0.1 C (red), 0.2 C (green) and 0.5 C (blue) rates.

Single-layer all-solid-state battery was prepared by stacking LiFePO_4_ cathode composite, quasi-soild-state electrolyte sheet and a Li metal anode. As shown in Figure 2, bipolar stacked all-solid-state batteries were fabricated by layering two or three single-layer batteries and trapping them with current collectors in the same CR2032 module of a coin cell. Figure 3 (a) shows the 10^th^ charge–discharge profiles of the single-layered (one cell unit), double-layered (two cell units) and triple-layered (three cell units) all-solid-state lithium batteries at 35°C and 0.1 C. For the single-layered battery, the 10^th^ discharge capacity was 161 mAh/g and the cathode utilization ratio was as high as 94%. For the double-layered and triple-layered batteries, the 10^th^ discharge capacities were 155 and 156 mAh/g, respectively, and the cathode utilization ratios were 91% and 91%, respectively. The cathode utilization ratios of the double-layered and triple-layered batteries were as high as that of the single-layered battery. The double-layered and triple-layered batteries showed plateau potentials of 6.7–6.8 and 10.0–10.2 V, respectively. These values for double-layered and triple-layered batteries were two and three times than that (3.4 V) of the single-layered battery, respectively, confirming that the electric cells within a single package did not have internal short-circuits and the stacked batteries operated successfully.

**Figure 4:**
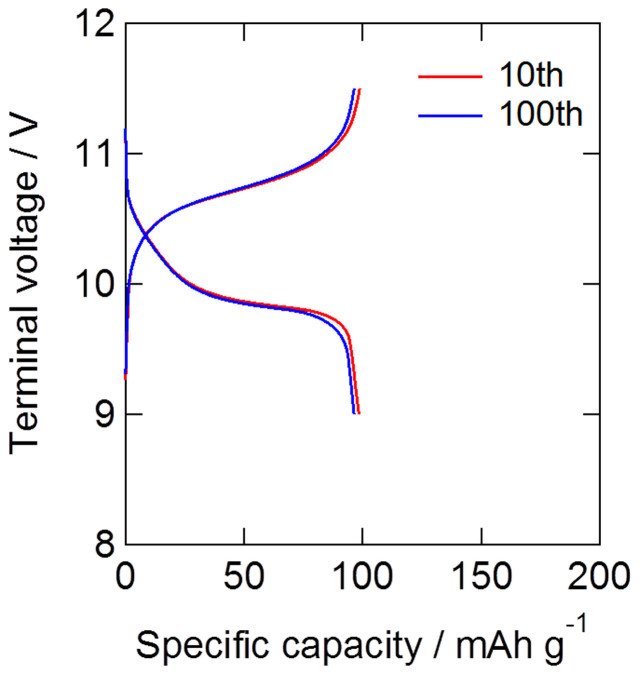
The 10^th^ and 100^th^ charge-discharge curves of triple-layered bipolar stacked cell at 35°C and 0.2 C.

Figure 3 (b) shows the charge–discharge profiles of the first and 100th cycles for the double-layered all-solid-state lithium battery at rates of 0.1 C, 0.2 C and 0.5 C. The initial capacities at 0.2 C and 0.5 C were 140 and 118 mAh/g, respectively, and plateau potentials were observed at 6.6–6.7 and 6.4–6.5 V, respectively. These results indicated that, at 0.2 C and 0.5 C, the double-layered battery showed a high cathode utilization ratio, i.e., similar to that of the single-layered battery, and showed that the device could be operated successfully without any internal short-circuiting. Figure 3 (c) shows the cycling properties and coulombic efficiencies of the double-layered all-solid-state device at 0.1 C, 0.2 C and 0.5 C. The coulombic efficiencies at 0.1 C, 0.2 C and 0.5 C were as high as 99, 98 and 99%, respectively. After the 200th cycle, the device retained 91, 89 and 99% of the capacity after the 10th cycle at each C-rate, respectively, indicating good cycling properties. Figure 4 shows the 10^th^ and 100^th^ charge–discharge profiles of the triple-layered all-solid-state lithium battery at rate of 0.2 C. The 10^th^ and 100^th^ discharge capacities were 98 and 96 mAh/g, respectively, indicating good cycle performance. Only a few studies on stacked all-solid-state devices have been reported to date and, although stacked devices using polymer electrolytes^3^,^5^ or Li_3_PO_4_^4^ have been investigated, they showed low cathode utilization ratios because of their high internal electrolyte resistance. Furthermore, there have been a few studies on evaluation of cycle property to date. Thus, to the best of our knowledge, this is the first report on stacked all-solid-state lithium batteries which indicates good cycle properties, and we successfully developed the high-voltage all-solid-state lithium devices in 6 and 10 V classes.

### Cross-sectional SEM image of all-solid-state Li cell

**Figure 5:**
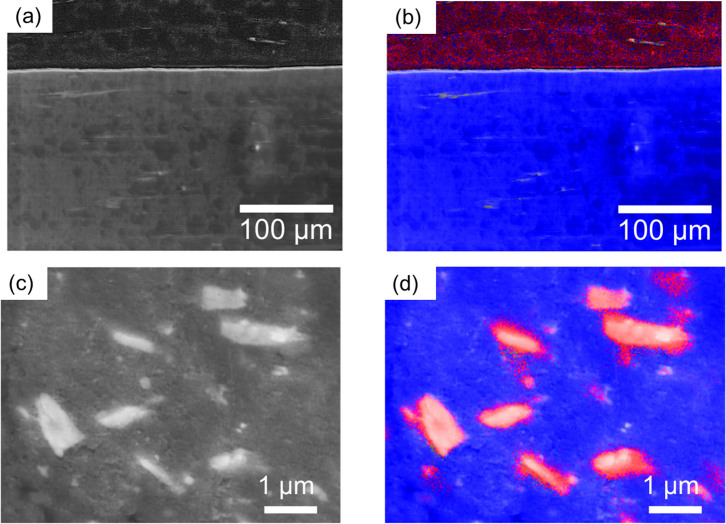
(a) Cross-sectional SEM image and (b) Si (blue) and P (red) distributions of the all-solid-state Li battery. (c) SEM image and (d) Si (blue) and P (red) distributions of the cathode composite containing LiFePO_4_ and quasi-solid electrolyte using fumed SiO_2_.

Figure 5(a) shows a cross-sectional SEM image of the all-solid-state lithium battery, and Figure 5(b) shows the EDX mapping results. The red region represents P derived from LiFePO_4_, and the blue region represents Si derived from fumed SiO_2_. This SEM image indicated the formation of a continuous and proper interface between the cathode and electrolyte. Furthermore, the SEM images of the cathode composite in Figures 5(c) and (d) confirmed the formation of a good lithium-ion transport interface between LiFePO_4_, and the quasi-solid-state electrolyte in the cathode composite, which led to the high efficiency of the cathode for the stacked all-solid-state batteries prepared in this study.

## Discussion

High-voltage stacked lithium batteries using quasi-solid-state electrolytes containing a Li-Glyme complex were prepared, and the performances of the devices were evaluated via charge–discharge testing. It was confirmed that no internal short-circuits occurred within the single packages containing double- and triple-layered devices, and these devices operated successfully. We successfully developed stacked lithium batteries with high cathode utilization ratio and good cycling properties.

## Methods

### Preparation of quasi-solid-state electrolyte

Equimolar amounts of lithium bis(trifluoromethanesulfonyl)amide powder (Li-TFSA,>99%, Kishida Chemical Co.) and tetraethylene glycol dimethyl ether (Tetraglyme, G4, 99%, Sigma-Aldrich Co.) were mixed to obtain a [Li(G4)][TFSA] solution. This [Li(G4)][TFSA] solution and fumed silica nanoparticles (Sigma-Aldrich Co., specific surface area: 390 m^2^ g^−1^, particle size: 7 nm) were then mixed and stirred in methanol for 3 h to achieve a volume fraction of 80%. The resulting mixture was dried on a hot plate at 60°C for 12 h to obtain a quasi-solid-state electrolyte powder. The resulting quasi-solid-state electrolyte powder and polytetrafluoroethylene (PTFE, Teflon-J, DuPont-Mitsui Fluorochemicals Co., Ltd.) were mixed to obtain a 5 wt% mixture, which was used to prepare a free-standing films with 200 μm thickness and 12 mm diameter.

### Performance evaluation of bipolar stacked all-solid-state lithium batteries

LiFePO_4_ (Tatung Fine Chemicals Co., theoretical capacity: 170 mAh/g), acetylene black (AB), the quasi-solid-state electrolyte powder, and PTFE were mixed (weight fraction of 34:11:45:10) and a cathode composite with cathode weight of 2 mg, 100 μm thickness and 4 mm diameter was prepared. A single-layered all-solid-state lithium secondary battery was prepared by directly stacking cathode composite, φ 12 mm quasi-solid-state electrolyte sheet with 200 μm thickness and φ 10 mm lithium metal anode with 100 μm thickness. As shown in Figure 2, two or three single-layered all-solid-state batteries were sandwiched between current collectors (SUS304L) and stacked in the same CR2032 module of a coin cell to prepare high-voltage stacked all-solid-state batteries. The cut-off voltage was set at 2.0–4.0 V for the single-layered battery, while those of the double-layered and triple-layered batteries were set at 5.0–8.0 V and 7.0–12.0 V, respectively. Charge–discharge measurements were performed on a HOKUTO Denko Corp. 8CH charge/discharge unit for the single-layered and double-layered batteries and a HOKUTO Denko Corp. HZ-7000 electrochemical measurement system for the triple-layered battery at 35 °C and 0.1 C and 0.2 C. The charge-discharge characteristics of double-layered cells were evaluated at 0.1 C, 0.2 C and 0.5 C.

### Cross-sectional SEM image of all-solid-state cell

A scanning electron microscope (SEM, SU-6600, HITACHI) and an energy dispersive X-ray spectrometer (EDX, Incas-act, Oxford Instruments) were used to characterize the cathode composite. Cross-section polisher (IB-09020CP, JEOL) was used to prepare cross-sectional all-solid-state device for observation.

## Author Contributions

Y.G. and I.H. conceived and designed this work. Y.G. and Y.S. carried out the fabrication of cells and conducted the electrochemical test. Y.G. wrote the paper; all the authors participated in analysis and discussion of the results.

## Figures and Tables

**Figure 1:**
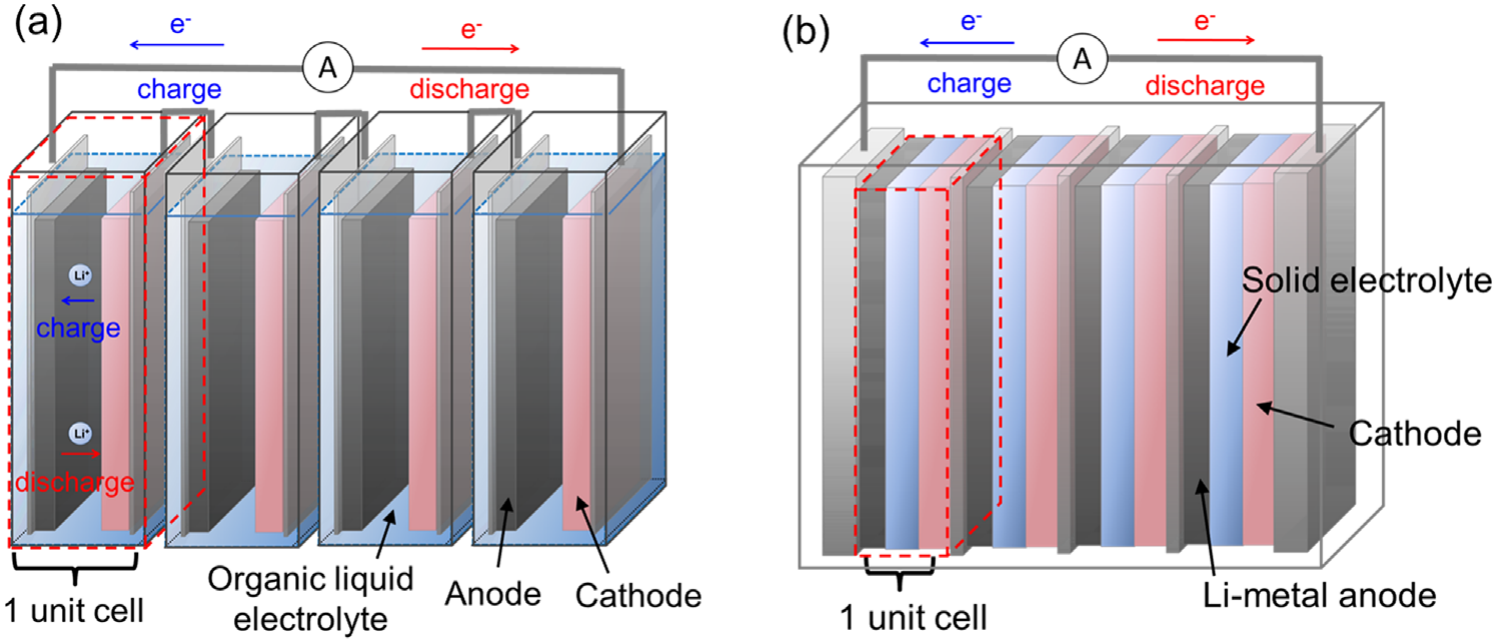
Schematic of (a) conventional stacked Li-ion battery using a liquid electrolyte and (b) bipolar stacked all-solid-state Li battery.
